# Notes and News

**Published:** 1895-08-03

**Authors:** 


### Notes and News.

(Collected and Communicated.)

A new and salutary bye-law has been introduced into the College of Surgeons which will enable the council to remove the name of any Fellow or member from its rolls who has been placed guilty of any serious charge in any professional respect. This will give the council a certain power over persons against whom, unfortunately, any such charges can be made.

The inaugural meeting of the Association of Asylum Workers, held at the rooms of the Medical Society, 11, Chandos Street, was presided over by Sir Benjamin Ward Richardson. The objects of this Association were pointed out by the Secretary as being the wish to improve generally the status of asylum nurses and attendants, to secure the sympathy and co-operation of all those interested in institutional work and efforts, and to provide a "home of rest and nursing" for those engaged in asylum work. No such home exists at present, and the need for it is a serious one, as the Secretary pointed out.

The Board of Superintendence of the Dublin Hospitals has just issued its thirty-seventh report. It shows that the year ended March 31st, 1895, was an exceptionally severe one for the Dublin hospitals, the city being visited with a small-pox epidemic, which necessitated the entire giving up of the Hardwicke Hospital for the treatment of these cases. The resources of the Cork Street Hospital were also much taxed, but the great difficulty experienced in stamping out the dread disease was found in the objection entertained by patients and their friends towards entering a hospital at all. This the board believes to be due to the stringent rules against visits of relatives to small-pox patients, and suggests that under proper precautions some relaxation might be effected. The average annual cost per bed among the hospitals generally is stated to be something over £60. It is satisfactory to see that a gradual improvement takes place year by year in the condition of the hospitals, and a greater desire is shown to bring them up to modern requirements.

The Yarrow Convalescent Home, opened at Broadstairs on Wednesday, has been erected through the munificence of Mr. Yarrow. It is intended for the reception of children of highly respectable parentage only, who will be required to pay 5s. weekly for the maintenance of the child and railway fares also. There is accommodation for one hundred children. The situation is admirable. The building, which is a handsome one, stands on the high Downs of Broadstairs, with an uninterrupted view of the sea, and a balcony runs nearly the whole length of the front.

The proceedings at the meeting of the Council of the Metropolitan Hospital Sunday Fund on Monday raised three interesting points. Twelve deputations from institutions seeking a grant had been received by the Distribution Committee, in response to questions which had been raised on their accounts and management. We direct special attention to the paragraph of the Distribution Committee's report, published in another column, which deals with this important portion of its work. Grants had been refused to certain institutions, including the Jubilee Hospital, which Mr. Burdett stated he had inspected, and found to be an overcrowded, insanitary tenement, where sick people were wrongly taken in for medical treatment. Dr. Glover called attention to the increasing number of out-patients admitted to hospitals receiving grants, and gave notice of a resolution to appoint a special committee to consider the questions involved.

At a meeting of the Metropolitan Hospital Sunday Fund Council on Monday last it was stated by Sir Sydney Waterlow that the receipts already amount to £44,000, that £2,000 is receivable, making a total of £46,000, which is the largest sum ever received in the history of the fund. In 1891 the total receipts amounted to upwards of £45,000, when they included a special donation of £5,000 from the Duke of Cleveland, and in 1894 to £43,680, when Mr. Whitaker's legacy of £5,000 was included. During the present year no extraordinary gifts have been received, so that the £46,000 raised represents an increase of about £8,000 over the contributions of any previous year. The accounts are never closed until the end of October, and we have reason to know that steps are being taken which will, we have little doubt, raise the collections in 1895 to £50,000. Hospital Sunday should and must depend for its success upon the contributions of the many. It is everybody's fund, and the most satisfactory feature of the special efforts made in London in the present year in regard to these collections is that the contributions from the churches show, as a rule, a steady increase in amount when compared with previous years. It is fitting that we should offer to the Lord Mayor and to Mr. W. J. Soulsby the cordial acknowledgments of all who have the best interests of the hospitals at heart. Both the Lord Mayor and Mr. Soulsby have this year worked zealously and continuously on behalf of the hospitals. They have gladly done everything in their power to win public sympathy and support, and every effort has been put forth to secure £50,000 for the hospitals through the Fund in 1895. We are especially glad to note the appreciation shown by the Council for the valuable statistical work so ably done for the Fund by Mr. R. A. Owthwaite, who, like Mr. Custance, the secretary, is untiring in his zealous discharge of the duties which devolve upon him.

